# Analysis of interstitial lung disease in pharmacovigilance databases: Coding challenges and interpretation biases—An update

**DOI:** 10.1002/bcp.70483

**Published:** 2026-02-15

**Authors:** Romane Freppel, Adeline Benis, Philippe Bonniaud, Jean‐Luc Faillie, Aurélie Grandvuillemin

**Affiliations:** ^1^ CHU Dijon Bourgogne, Centre Régional de Pharmacovigilance de Bourgogne Université Bourgogne Europe Dijon France; ^2^ IDESP, Université de Montpellier, INSERM Montpellier France; ^3^ CHU Dijon Bourgogne, Institut Universitaire du Poumon, Centre Constitutif Maladies Pulmonaires Rares de l'Adulte, Inserm 1231 CTM Université Bourgogne Europe Dijon France; ^4^ Service de Pharmacologie Médicale et Toxicologie, Centre Régional de Pharmacovigilance CHU Montpellier Montpellier France

**Keywords:** adverse effect, database, disproportionality analysis, interstitial lung disease, pharmacovigilance, risk assessment

## Abstract

**Aim:**

Clinically, interstitial lung disease (ILD) is a heterogeneous group of respiratory disorders. Due to their low incidence, pharmacovigilance database analysis is useful to detect them. Precise diagnosis is challenging as well as coding in these databases. Query criteria are among the key elements for a good signal detection. This study aimed to investigate interpretation biases related to ILD coding in PV databases.

**Methods:**

MedDRA Preferred Terms (PT) included in the broad Standardized MedDRA Query (SMQ) ‘ILD’ for the top five known pneumotoxic drugs was described in VigiBase by reporting year (2000–2024), country, and reporter type. Then, a systematic literature review was conducted to identify PV studies using disproportionality analyses to detect drug‐induced ILD reporting signals, assessing query strategies, analysis units, and bias consideration.

**Results:**

The most frequently implicated known pneumotoxic drugs were amiodarone, methotrexate, nivolumab, pembrolizumab, and everolimus. On average per year, the PTs ‘ILD’ represented <40% of all PTs in the broad SMQ ‘ILD’. Terminology evolution (e.g., emergence of ‘immune‐mediated lung disease’ after 2019) and national preferences (France/Japan *vs*. USA/UK/Germany) were observed. Among 22 reviewed accessible studies, 50% used PT‐based queries, only 27% performed sensitivity analyses, and none discussed the impact of MedDRA coding evolution on analysis reliability.

**Discussion/Conclusion:**

Our analysis highlights the substantial variability in MedDRA coding that may affect the reliability of drug safety analyses. Moreover, studies conducted on PV databases do not sufficiently assess the relevance of query criteria and their impact on the results. We propose a few practical recommendations.

**What is already known about this subject**

- Interstitial lung diseases (ILDs) are heterogeneous lung disorders with variable clinical, radiological, and histopathological features, making accurate diagnosis challenging; drug‐induced ILD is an important but often underrecognized cause.
- Pharmacovigilance is essential for detecting rare, serious, or delayed‐onset drug‐induced ILD, but MedDRA coding variability, terminology complexity, and frequent updates complicate signal detection and case identification.
- Disproportionality analyses using large pharmacovigilance databases are key tools for identifying potential drug‐induced ILD reporting signals, but their reliability depends heavily on accurate clinical diagnosis, consistent MedDRA coding, and expert interpretation.

**What this study adds**

- The way ILD is coded in pharmacovigilance databases varies greatly across time, countries, and reporter types, creating inconsistencies that can distort disproportionality signal detection.
- Rare and complex drug‐induced ILD events are especially vulnerable to misclassification, which may compromise regulatory decisions.
- Our study highlights these challenges and provides practical recommendations to improve coding, query strategies, and analysis robustness for more reliable pharmacovigilance assessments.

## 1 INTRODUCTION

Interstitial lung diseases (ILDs) include a heterogeneous group of over 150 pulmonary disorders characterized by diffuse parenchymal lung involvement. Radiologically, ILDs typically present as bilateral, nonsystematized infiltrative opacities, while histopathologically, they involve cellular, edematous, or fibrotic infiltration of the pulmonary interstitium. Clinically, ILDs often manifest with nonspecific symptoms such as progressive dyspnoea, dry cough, fatigue, hypoxaemia, and inspiratory crackles. Given their variable presentations and diverse etiologies, diagnosing ILDs frequently requires a multidisciplinary approach integrating detailed clinical history, high‐resolution computed tomography, pulmonary function tests, and laboratory investigations. No single clinical, radiological, or histological pattern is pathognomonic for a specific ILD, making accurate diagnosis particularly challenging. Causes are multifactorial and include connective tissue diseases, infections or environmental exposure. Among these, drug‐induced ILD is an important but often underrecognized cause. To date, nearly 400 drugs have been implicated in ILD development according to the Pneumotox® website (www.pneumotox.com), and this number continues to grow. Diagnosing drug‐induced ILD is complex due to the absence of specific clinical or pathological features distinguishing it from other ILDs or attributing it to a particular drug. Common patterns associated with drug‐induced forms include pulmonary fibrosis, nonspecific interstitial pneumonia, hypersensitivity pneumonitis, and organizing pneumonia. The incidence of drug‐induced ILD is difficult to ascertain precisely but has been estimated between 4.1 and 12.4 cases per million per year. In ILD registries and cohorts, drug‐induced cases account for approximately 3% to 5% of all diagnoses.^1^, ^2^, ^3^ Pharmacovigilance plays a crucial role in the detection and evaluation of rare, serious, and often delayed‐onset adverse drug reactions (ADRs), such as drug‐induced ILD. Given their diagnostic challenges and nonspecific clinical manifestations, pharmacovigilance systems are essential for identifying potential drug‐related signals. Effective signal detection, however, relies heavily on accurate clinical diagnosis and consistent terminology coding in pharmacovigilance databases. Historically, the coding of ILD‐related adverse events was hampered by limitations in the terminology systems used. Prior to the widespread adoption of the Medical Dictionary for Regulatory Activities (MedDRA) in the mid‐2000s, pharmacovigilance databases used the WHO Adverse Reaction Terminology (WHO‐ART), which lacked granularity and semantic clarity. The term ‘interstitial pneumonia’ was ambiguously used, sometimes referring to atypical infections and sometimes to noninfectious interstitial lung disorders such as pulmonary fibrosis. WHO‐ART did not adequately link related terms such as ‘fibrosis’ and ‘interstitial pneumonia’, further complicating case classification and data interpretation. A 2005 study highlighted major geographical discrepancies in terminology usage.^4^ For example, France and Japan predominantly coded ILD cases under ‘interstitial pneumonia’ and rarely under ‘pneumonia’, whereas English‐speaking countries (e.g., United States of America [USA] and United‐Kingdom [UK]) tended to use ‘pneumonia’ more broadly. Today, MedDRA has become the internationally accepted standard for regulatory communication within the European Union, the USA, and Japan. With over 80 000 terms, MedDRA aims to promote semantic interoperability and facilitate robust signal detection in pharmacovigilance databases. ILDs are comprehensively captured in MedDRA through both clinical and radiologic definitions. The term ‘interstitial lung disease’ exists as a Preferred Term (PT) under the High‐Level Group Term (HLGT) ‘Lower respiratory tract disorders (excluding obstruction and infection)’, and refers to a single medical concept within the hierarchy. However, other ILD‐related entities—such as pulmonary fibrosis, organizing pneumonia, or hypersensitivity pneumonitis—are also listed as distinct PTs, even though they are part of the same clinical spectrum. To aid in the identification of ILD‐related cases, MedDRA includes a Standardized MedDRA Query (SMQ) for ILD. This SMQ includes 47 PTs under a narrow scope and an additional 36 PTs under a broad scope, offering a structured framework for case retrieval and analysis. Despite these advances, clinicians and pharmacovigilance professionals continue to face practical challenges when using standardized terminologies. Issues such as synonymy, polysemy, inconsistent classification, incomplete coverage, and term duplication can lead to coding discrepancies. Moreover, frequent updates to MedDRA (every 6 months) and ongoing evolution of the clinical definitions of ILD add further complexity. Among these, the case/noncase approach has become a widely used tool for investigating rare adverse events (AEs), capturing real‐world drug exposure and identifying safety signals. This methodology, however, is entirely dependent on accurate and consistent MedDRA coding. For complex conditions such as ILDs, both the clinical diagnosis and subsequent coding in pharmacovigilance databases pose significant challenges. This complexity is especially concerning in the context of the increasing use of large, open‐access pharmacovigilance databases for disproportionality analyses. While these analyses can generate valuable hypotheses, they are increasingly performed by nonspecialists who may lack specific training in pharmacovigilance methodology and its nuanced interpretation. As the number of such publications rises, so does the risk of methodological shortcuts, semantic misclassification, and misinterpretation of findings. In an era of rapid information dissemination—and sometimes misinformation—ensuring scientific rigour and transparency in pharmacovigilance research is essential. Disproportionality analyses, in particular, carry the potential to shape both public perception and clinical decision‐making, highlighting the critical importance of sound methodology and cautious interpretation.

### 1.1 Objectives

Twenty years after the introduction of MedDRA, this study aims to use ILD as a case example to examine the ongoing challenges in coding complex ADRs. The primary objective is to evaluate the diversity and specificity of MedDRA PTs used within the broad SMQ ‘Interstitial Lung Disease’ for the five drugs most frequently associated with ILD reports since 2000, based on data from the global pharmacovigilance database VigiBase. The secondary objective is to assess the methodology used in published disproportionality analyses involving ILD. Specifically, we aim to examine how authors construct MedDRA queries for case identification and whether they adequately address potential biases and limitations in the interpretation of their findings.

## 2 METHODS

### 2.1 VigiBase‐descriptive analysis

#### 2.1.1 Study design and data source

We conducted a retrospective descriptive study using data from the World Health Organization (WHO) global pharmacovigilance database, VigiBase. Maintained by the Uppsala Monitoring Centre (UMC), VigiBase has collected reports of ADRs from approximately 150 countries since 1968 and is considered a global reference for pharmacovigilance research. As of March 2024, the database contained over 38 million Individual Case Safety Reports (ICSRs). All ADRs in VigiBase are coded using the Medical Dictionary for Regulatory Activities (MedDRA®), an internationally standardized hierarchical terminology with five levels: Lowest Level Terms (LLTs), Preferred Terms (PTs), High‐Level Terms (HLTs), High‐Level Group Terms (HLGTs), and System Organ Classes (SOCs). Additionally, Standardized MedDRA Queries (SMQs)—predefined sets of terms relevant to key pharmacovigilance topics—are available in both narrow and broad formats for optimized case retrieval.

#### 2.1.2 Data extraction, processing, and analysis

Data were extracted from VigiBase via the VigiLyze interface on 10 March 2024.

#### 2.1.3 Selection criteria

We included all ICSRs involving at least one PT contained in the broad SMQ for ‘Interstitial Lung Disease’ to capture the widest range of relevant terms (a total of 83 PTs, detailed in Table S1). We focused on the five drugs most frequently associated with ILD in VigiBase, based on known risks listed in their respective Summaries of Product Characteristics (SmPCs). Reports were excluded if they contained PTs not related to ADRs (e.g., ‘Transfusion‐related acute lung injury’, ‘e‐cigarette or vaping product use associated lung injury’), if they were considered potential duplicates, or if they were related to vaccines, which are known to generate large, stimulus‐driven reporting waves that can mask signals. Duplicate detection was conducted using a two‐step approach, combining automated identification via the VigiMatch algorithm^5^ with manual validation. The detailed methodology and criteria used for identifying and resolving duplicates are fully described in Supporting Information S2.

#### 2.1.4 Statistical analysis

Following data cleaning and categorization, we conducted descriptive analyses based on three key parameters, each mandatory in VigiBase entries. The first parameter was the year of report (based on the ‘National PV Centre latest receive date’), analysed from 2000 onwards in 5‐year intervals. For immune checkpoint inhibitors, introduced after 2015, we used 2‐year intervals through March 2024. The second parameter was the reporting country, restricted to the five countries with the highest number of reports: France, Germany, Japan, the United Kingdom, and the United States. The third parameter concerned the type of reporter, categorized as healthcare professional or nonhealthcare professional.

We analysed the PTs that represented approximately 90% (±1%) of all coded terms between 2000 and 2024. The remaining terms were grouped under a generic ‘Other’ category. Categorical variables such as age, sex, report year, type of reporter, and reporting region were summarized as percentages. Figures and tables were generated using Microsoft Excel and Python. All statistical analyses were performed using R software.

### 2.2 Systematic review of the literature

#### 2.2.1 Study design and data source

We conducted a systematic review of the literature focusing on pharmacovigilance‐based studies of drug‐induced ILD using disproportionality analyses. Searches were performed in PubMed (https://pubmed.ncbi.nlm.nih.gov) up to July 2025. This review was conducted in accordance with the PRISMA 2020 guidelines for systematic reviews.^6^ The protocol was registered on PROSPERO under the registration number CRD420251108716.

#### 2.2.2 Data extraction, processing, and analysis

The following keyword combinations were used:

- (interstitial lung disease) AND (drug induced) AND (pharmacovigilance)
- (interstitial lung disease) AND (drug induced) AND (VigiBase)
- (drug induced interstitial lung disease) AND (disproportionality analysis)
- (lung toxicity) AND (drug induced) AND (pharmacovigilance)

Additional references were identified through manual screening of citation lists from relevant studies. The selection was performed in two phases. Two reviewers (R.F. and A.G.) independently screened titles and abstracts in a blinded manner. In cases of disagreement, a third reviewer (JL.F.) was consulted to reach consensus. Full‐text review was then conducted for articles deemed potentially eligible.

#### 2.2.3 Selection criteria

Only studies published in English were included. Studies were excluded if they did not involve a disproportionality analysis, if they focused on pharmacogenetics, on the clinical management of pulmonary toxicity or if they did not focus on drug‐induced pulmonary toxicity.

#### 2.2.4 Data analyses

From each included study, we extracted information on the drug of interest, the MedDRA coding strategy used (e.g., PT, SMQ, HLT, and SOC), as well as the specifications of the disproportionality method, including the analysis unit and any sensitivity analyses performed. We also assessed the quality of the discussion and whether potential sources of bias—such as coding variability, reporter type, or geographical differences—were discussed.

### 2.3 Ethical considerations

Ethics approval was not required for this study, as it was retrospective in nature and based solely on anonymized data.

This article does not describe or investigate specific protein targets or ligands. Consequently, no hyperlinks to the Guide to PHARMACOLOGY 2021/22 are included.

## 3 RESULTS

### 3.1 Evolution of MedDRA coding of preferred terms within the broad SMQ ‘interstitial lung diseases’

#### 3.1.1 Descriptive analysis of included ILD ICSRs

**FIGURE 1:**
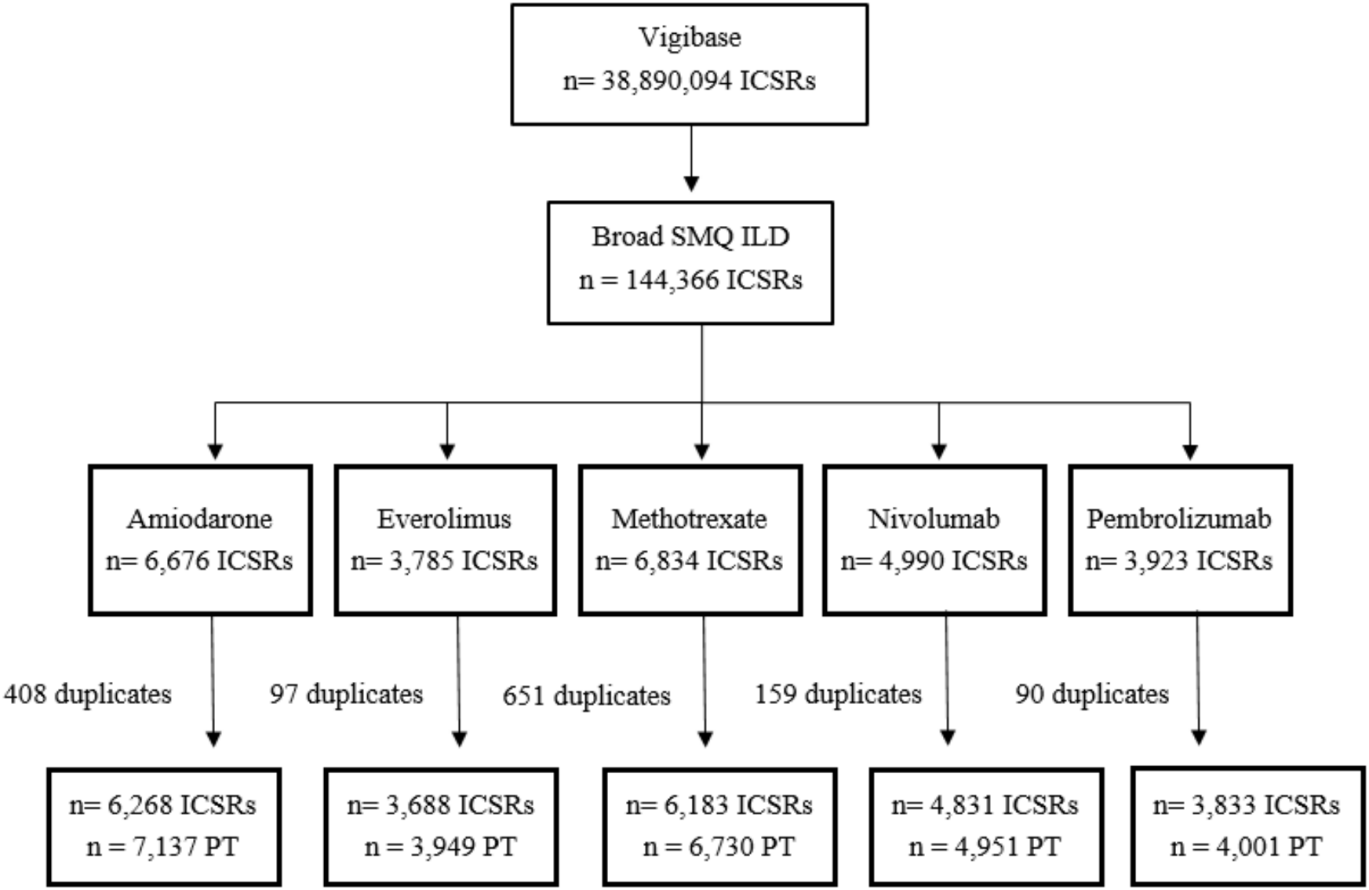
Study subject inclusion flowchart.

Figure 1 presents the flow diagram summarizing the inclusion strategy. As of March 2024, a total of 144 366 individual case safety reports (ICSRs) were retrieved using the broad SMQ ‘interstitial lung disease’ in the VigiBase database. The most frequently implicated known pneumotoxic drugs were amiodarone (6268 ICSRs, 4.3%), methotrexate (6183 ICSRs, 4.2%), nivolumab (4831 ICSRs, 3.3%), pembrolizumab (3833 ICSRs, 2.6%), and everolimus (3688 ICSRs, 2.6%).

Table 1 presents the patients characteristics for ILD cases reported with each of the five drugs. Table S3‐1 differentiates between cases where the drug was the sole suspected agent and those where it was reported as a co‐suspect. Drugs were reported as the sole suspect in varying proportions: amiodarone (71.6%), methotrexate (43.0%), everolimus (63.4%), pembrolizumab (68.0%), and nivolumab (60.9%). The sex distribution varied by drug and whether it was reported as the sole suspected drug or as a co‐suspect (Table S3‐1). A higher proportion of male patients was observed with amiodarone (65.8%) and nivolumab (65.4%). Conversely, females were more represented in methotrexate and everolimus‐related ILD. Sex was not reported in 2%–8% of cases, depending on the drug. The median age of patients ranged between 63 and 75 years. Amiodarone‐related ILD involved the oldest patients (median: 75 years), whereas methotrexate and everolimus were associated with younger cases (median: 64 and 63 years, respectively).

**TABLE 1:** Main characteristics of patients with ILD associated with five drugs.

Characteristics (*n*, %)	Amiodarone	Everolimus	Methotrexate	Nivolumab	Pembrolizumab
	*N* = 6268	*N* = 3688	*N* = 6183	*N* = 4831	*N* = 3833
Male	4127 (65.8)	1108 (30.0)	2023 (32.7)	3159 (65.4)	2480 (64.7)
Female	1940 (31.0)	2313 (62.7)	3770 (61.0)	1269 (26.3)	1143 (29.8)
Not available	201 (3.2)	267 (7.2)	390 (6.3)	403 (8.3)	209 (5.5)
**Age (*n*, %)**					
Mean	73.1	62.6	61.5	64.6	66.5
Median (Q1; Q3)	74 (67; 80)	64 (56; 71)	65 (56; 73)	67 (59; 73)	69 (61; 75)
Min; max	1; 99	0; 100	0; 95	0; 94	4; 100
	*n* = 4962 (79.2)	*n* = 2366 (64.2)	*n* = 4550 (73.6)	*n* = 3799 (78.6)	*n* = 3071 (80.1)

The majority of cases were serious, with high proportions of hospitalization (31%–42%), life‐threatening events (5%–10%), or death (7%–21%). Notably, death was more frequently reported in co‐suspect cases of amiodarone (21.6%). In terms of clinical outcomes, around one third of the cases were recovering or recovered, and up to 17% had a fatal outcome, particularly for amiodarone and immune checkpoint inhibitors. A notable proportion of outcomes was missing or classified as ‘unknown’, reaching up to 30% in some drug groups. The most frequently coded MedDRA Preferred Term (PT) across all drugs was ‘interstitial lung disease (ILD)’, as shown in Table 2. Other recurrent terms included pneumonitis, pulmonary fibrosis, and pulmonary toxicity. Immune‐mediated lung disease was reported exclusively in association with immune checkpoint inhibitors (nivolumab and pembrolizumab).

**TABLE 2:** Characteristics of ILD‐related cases reported with amiodarone, everolimus, methotrexate, nivolumab and pembrolizumab.

Characteristics (*n*, %)	Amiodarone	Everolimus	Methotrexate	Nivolumab	Pembrolizumab
	*N* = 7137	*N* = 3949	*N* = 6730	*N* = 4951	*N* = 4001
**MedDRA preferred term of the ILD broad SMQ**					
Interstitial lung disease	2197 (30.8)	1581 (40.0)	2421 (36.0)	1803 (36.4)	1368 (34.2)
Pulmonary fibrosis	1942 (27.2)	68 (1.7)	1043 (15.5)	40 (0.8)	63 (1.6)
Pulmonary toxicity	854 (12.0)	74 (1.9)	209 (3.1)	43 (0.9)	46 (1.2)
Pneumonitis	554 (7.8)	1678 (42.5)	1170 (17.4)	2088 (42.2)	1601 (40.0)
Lung infiltration	370 (5.2)	176 (4.5)	321 (4.8)	38 (0.8)	38 (1.0)
ARDS	334 (4.7)	53 (1.3)	403 (6.0)	55 (1.1)	49 (1.2)
Organizing pneumonia	167 (2.3)	66 (1.7)	132 (2.0)	165 (3.3)	118 (3.0)
Alveolitis	113 (1.6)	37 (1.0)	123 (1.8)	62 (1.3)	7 (0.2)
Pulmonary alveolar haemorrhage	121 (1.7)	13 (0.3)	31 (0.5)	16 (0.3)	11 (0.3)
Immune‐mediated lung disease	0 (0)	0 (0)	0 (0)	363 (7.3)	336 (8.4)
Other	485 (6.8)	203 (5.1)	877 (13.0)	278 (5.6)	364 (9.1)
**Seriousness**					
Serious	5338 (74.8)	3204 (81.2)	5528 (82.1)	4625 (93.4)	3682 (92.0)
Not serious	1 (<0.1)	2 (<0.1)	15 (<0.1)	6 (<0.1)	0 (0)
Not available	1798 (25.2)	743 (18.8)	1187 (17.6)	320 (6.5)	319 (8.0)
**Outcome**					
Died	1197 (16.8)	176 (4.5)	696 (10.3)	496 (10.0)	451 (11.3)
Not recovered	1091 (15.3)	290 (7.3)	892 (13.3)	455 (9.2)	457 (11.4)
Recovering	1142 (16.0)	734 (18.6)	891 (13.2)	1150 (23.2)	936 (23.4)
Recovered	927 (13.0)	1219 (30.9)	1281 (19.0)	1133 (22.9)	839 (21.0)
Recovered with sequelae	203 (2.8)	56 (1.4)	118 (1.8)	91 (1.8)	48 (1.2)
Unknown	1473 (20.6)	1164 (30.0)	2134 (31.7)	1511 (30.5)	1122 (28.0)
Not available	1104 (15.5)	310 (7.9)	705 (10.5)	109 (2.2)	148 (3.7)
**Number of suspected drugs per case**	3.1	3.1	4.4	3.0	3.8
Reporting year					
<2000	3 (<0.01)	2 (0.05)	9 (0.1)	0	0
2000–2004	342 (4.8)	0	214 (3.2)	0	0
2005–2009	817 (11.5)	69 (1.8)	589 (8.8)	0	0
2010–2014	1110 (15.6)	1252 (31.7)	1345 (20.0)	2	0
2015–2019	2095 (29.4)	2165 (54.8)	2597 (38.6)	/	/
2015–2016	/	/	/	596 (12.0)	117 (3.0)
2017–2018	/	/	/	1902 (38.4)	1256 (31.4)
2019–2020	/	/	/	1209 (24.4)	1187 (30.0)
2020–2024	1167 (16.4)	361 (9.1)	1006 (15.0)	/	/
2021–2022	/	/	/	840 (17.0)	816 (30.4)
2023–2024	/	/	/	362 (7.3)	584 (14.6)
Not available	1603 (22.5)	100 (2.5)	957 (14.2)	40 (0.8)	41 (1.0)
**Reporter**					
Healthcare professionals	4541 (63.6)	3013 (76.3)	4600 (68.4)	3471 (70.1)	3099 (77.5)
Nonhealthcare professionals	1706 (23.9)	791 (20.0)	1462 (21.7)	1424 (28.8)	822 (20.5)
Not available	890 (12.5)	145 (3.7)	668 (9.9)	56 (1.1)	80 (2.0)
**Country of primary source**					
France	1472 (20.6%)	241 (6.1%)	502 (7.5%)	443 (9.0%)	346 (8.7%)
Germany	411 (5.8%)	485 (12.3%)	386 (5.8%)	354 (7.2%)	239 (6.0%)
UK	408 (5.7%)	100 (2.5%)	450 (6.7%)	39 (1.4%)	129 (3.2%)
Japan	477 (6.7%)	1099 (27.8%)	1233 (18.4%)	1730 (35.0%)	1165 (29.1%)
USA	2614 (36.6%)	774 (19.6%)	1975 (29.4%)	1134 (22.9%)	825 (20.6%)
Other	1755 (24.6%)	1250 (31.7%)	2184 (32.3%)	1251 (25.0%)	1297 (32.4%)

Abbreviations: ARDS, Acute Respiratory Distress Syndrome; UK, United Kingdom; USA, United States of America.

When available, time to onset varied widely, from day 0 to over 20 years for drugs like amiodarone and methotrexate, while for immunotherapies, onset generally occurred within 3 to 7 years. Co‐suspect reports involved an average of three to four suspected drugs per case, frequently including prednisone/prednisolone, lenvatinib, ipilimumab, and carboplatin in nivolumab and pembrolizumab cases, and biologic DMARDs (Disease‐Modifying Anti‐Rheumatic Drugs) in methotrexate‐associated ILD (Table S3‐2).

#### 3.1.2 Distribution of MedDRA coding by time, reporter type, and country

##### Temporal trends in reporting

**FIGURE 2:**
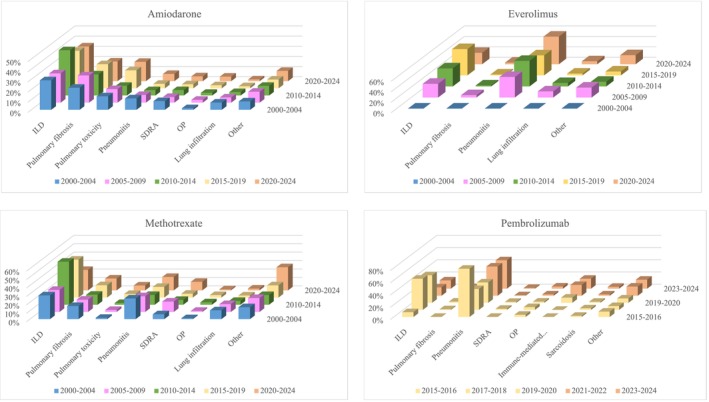
Temporal trends in preferred term coding within the broad SMQ ‘ILD’.

The number of PTs within the broad SMQ ‘Interstitial Lung Disease’ (ILD) increased progressively over time, particularly between 2010 and 2024 for all five drugs. For amiodarone, methotrexate, and everolimus, reports began earlier and peaked between 2015 and 2019, while for nivolumab and pembrolizumab, a sharp rise was observed from 2015 onwards, corresponding with the growing use of immune checkpoint inhibitors. Notably, for nivolumab, 38.4% of reports occurred in 2017–2018, and for pembrolizumab, 31.4% were concentrated between 2017 and 2018 (Table 2, Figure 2).

##### Reporter type and reporting characteristics

The majority of reports originated from healthcare professionals, ranging from 70.1% (nivolumab) to 79.2% (everolimus). Nonhealthcare professionals accounted for 20%–30%, with a substantial proportion of missing data, especially for amiodarone (13% of cases had unknown reporter type) (Table 2).

##### Geographical patterns

**FIGURE 3:**
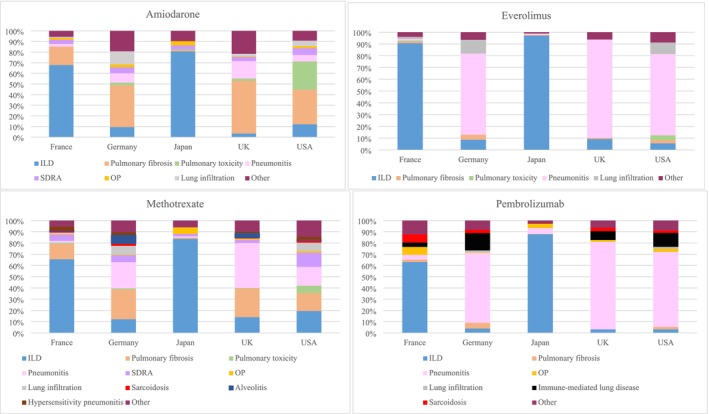
Geographical distribution of preferred term coding within the broad SMQ ‘ILD’ by top five reporting countries.

Reports predominantly originated from the USA (19.6%–36.6%), France (6.1%–20.6%), and Japan (18.4%–35.0%). Marked terminological heterogeneity in PT coding was noted. On average, the PT ‘interstitial lung disease’ accounted for less than 40% of all PTs within the ILD SMQ. Since 2019, coding patterns for immunotherapy‐related ILD have shifted, with emergence of ‘immune‐mediated lung disease’, which represented up to 30% of PTs for nivolumab and pembrolizumab. In France and Japan, ‘interstitial lung disease’ was the dominant PT (55%–96% of cases), while in the USA and Germany, the term ‘pneumonitis’ was more frequently used (Table 2, Figure 3).

The analyses revealed clear differences in PT selection by reporter type, documented in Table S3‐3. For instance, ‘pneumonitis’ was much more frequently reported by nonhealthcare professionals, especially for nivolumab (63.1% of non‐HCP reports), whereas ‘interstitial lung disease’ and ‘pulmonary fibrosis’ were dominant among healthcare professionals. The recently introduced PT ‘immune‐mediated lung disease’ was reported almost exclusively by healthcare professionals.

### 3.2 Literature review

**FIGURE 4:**
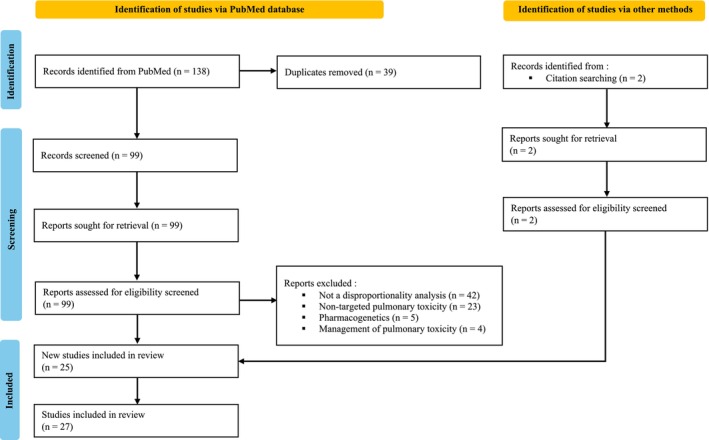
Study identification flow chart.

Among 138 publications retrieved, we included 27 publications investigating the association between drugs and ILD using pharmacovigilance databases (Figure 4). There was a clear upward trend in the number of studies employing disproportionality analyses, with the majority published after 2020. The most frequently used databases were FAERS (*n* = 15), VigiBase (*n* = 6), JADER (*n* = 5), and Eudravigilance (*n* = 1) (Table 3).

**TABLE 3:** Methodological approaches.

Author and year of publication	Pharmacovigilance database	Drug of interest	Specification of the method use		
			MedDRA query method	Specification of the disproportionality analysis unit	Sensitivity analyses
Matsumoto K et al., 2020^7^	JADER	All drugs	PT ‘interstitial lung disease’	No	No
Sato et al., 2022^8^	JADER	Alectinib	Not specified	No	No
Kanbayashi Y et al., 2022^9^	JADER	Trastuzumab	Not specified	‘“Cases” indicates the number of reported cases of pulmonary toxicity’	No
Kozaru et al., 2024^10^	JADER	Clopidogrel	PT ‘interstitial lung disease’	‘The number of reports of a specific AE’	No
Oura K et al., 2024^11^	JADER	Herbal medicines	PT ‘interstitial lung disease’	‘a = the number of AE reports in which patients received the herbal medicine and manifested drug induced interstitial lung disease’	No
Oshima et al., 2018^12^	FAERS	EGFR‐ITK + nivolumab	Not specified	‘Patients with interstitial pneumonitis’	No
Raschi E et al., 2020^13^	FAERS	NOAC	PT ‘interstitial lung disease’	No	Only suspects Exclude Japanese cases Exclude co‐suspected pneumotoxic drugs Exclude ICSRs with excessive adverse events
Raschi E et al., 2021^14^	FAERS	Anti‐CDK4‐6	PT ‘interstitial lung disease’	No	Only suspects Exclude Japanese cases Exclude co‐suspected pneumotoxic drugs Exclude ICSRs with excessive adverse events
Goldman A et al. 2021^15^	FAERS	Chimeric antigen receptor T‐cell	PTs: ‘pneumonitis’, ‘Hypersensitivity pneumonitis’, ‘immune‐mediated pneumonitis’, ‘acute interstitial pneumonitis’, ‘pneumonitis chemical’, ‘granulomatous pneumonitis’ Narrow SMQ ‘interstitial lung disease’	‘*N* observed = the number of case reports for the drug‐AE combination’	Exclude cases with the indication ‘B‐cell acute lymphoblastic leukaemia’
Wu B et al., 2022^16^	FAERS	Hormone therapy	SMQ narrow ‘interstitial lung disease’	‘ILD event’	No
Zhao et al., 2023^17^	FAERS	ITK	?	?	?
Zhang J et al., 2023^18^	FAERS	ITK	?	?	?
Shi J et al., 2024^19^	FAERS	Anti‐body drug conjugates	Broad SMQ ‘interstitial lung disease’	No	No
Lerman TT et al., 2024^20^	FAERS	Amiodarone	PT ‘interstitial lung disease’	No	No
Lin W et al., 2024^21^	FAERS	Anti‐body drug conjugates	Not specified	‘Cases were defined as instances where “interstitial lung disease” was recorded for a given drug’	No
Jiang T et al., 2025^22^	FAERS	All drugs	PT ‘interstitial lung disease’	‘Number of reports with drug induced interstitial lung disease caused by the target drug’	No
Shi J et al., 2025^23^	FAERS	EGFR–TKI	PTs: ‘pneumonia’, ‘interstitial lung disease’, ‘respiratory failure’, ‘pleural effusion’ SOC: ‘respiratory thoracic mediastinal disorder’	No	No
Yuan J et al., 2025^24^	FAERS	All drugs	?	?	?
Wang H et al., 2025^25^	FAERS	Combination of Proton Pump Inhibitors and EGFR‐TKI	Narrow SMQ ‘interstitial lung disease’	‘Interstitial lung disease event’	Excluded East Asian populations
Mo Y et al., 2025^26^	FAERS	Immune checkpoint blockade combined with radiotherapy	PT ‘pulmonary toxicity’, ‘idiopathic pulmonary fibrosis’, ‘interstitial lung disease’, ‘pneumonitis chemical’, ‘pneumonitis’, ‘pulmonary fibrosis’, ‘radiation pneumonitis’	‘Cases were identified as reports of pulmonary AEs’	No
Reese SW et al., 2021^27^	VigiBase	Immune checkpoint inhibitors	20 PT categorized into 6 groups based on expert consensus (interstitial, vascular, pleural, alveolar, airway, nonspecific)	‘Pulmonary toxicity events’	No
Ma Z et al., 2022^28^	VigiBase	Anti‐HER2 ADCs	PT ‘interstitial lung disease’, ‘pneumonitis’	?	?
Ma Z et al., 2022^29^	VigiBase	EGFR/ALK TKIs	PT ‘interstitial pneumonitis’	?	?
Lee MT et al., 2023^30^	VigiBase	COVID‐19 vaccines	Narrow SMQ ‘interstitial lung disease’	‘ICSR’	‐Vaccines: only suspected *vs*. concomitant/interaction ‐Serious AE reports only ‐Broad SMQ ‘interstitial lung disease’ ‐Exclusion of pneumotoxic co‐suspect drugs ‐Exclusion of ICSR reports included COVID‐19 vaccine
Givry et al., 2025^31^	VigiBase	Cotrimoxazole	HLT ‘Lower respiratory tract disorders’	‘The proportion of lung toxicity reported’	Grouped similar lung toxicity patterns: ‐ ‘interstitial lung disease’ = ‘interstitial lung disease + lung infiltration’ ‐ ‘eosinophilic pneumonia’ = ‘pulmonary eosinophilia + eosinophilic pneumonia + eosinophilic pneumonia acute’ ‐ ‘pneumonitis’ = ‘pneumonitis + acute interstitial pneumonitis’
Minagi A et al., 2025^32^	VigiBase	Biologic agents	Narrow SMQ ‘interstitial lung disease’	‘a = number of patients with “interstitial lung disease” administered each biologic agent’	No
Pinheiro L et al., 2016^33^	Eudravigilance	All drugs	PT ‘interstitial lung disease’ PT ‘pulmonary fibrosis’ SOC ‘respiratory, thoracic and mediastinal disorders’	‘Interstitial lung disease’ cases	No

Abbreviations: ADC, human epidermal growth factor receptor 2 antibody‐drug conjugate; ALK, anaplastic lymphoma kinase; Anti‐CDK4‐6, cyclin‐dependent kinase 4 and 6 inhibitor; EGFR, epithelial growth factor receptor; FAERS, FDA Adverse Event Reporting System; JADER, Japanese Adverse Drug Event Report database; NOAC, non‐vitamin K oral anticoagulant; TKI, tyrosine kinase inhibitor.

#### 3.2.1 Methodological approaches

As shown in Table 3, among the accessible articles (22/27), most studies (11/22) relied on MedDRA queries based on Preferred Terms (PTs), often limited to terms such as ‘interstitial lung disease’. Four articles did not clearly describe their query strategies, limiting the reproducibility of the analyses (Figure S4). When specified (15/22), the analysis unit used in disproportionality assessments varied across studies: Five (22%) were based on the number of events, 10 (45%) on individual case safety reports (ICSRs). Only six studies performed sensitivity analyses (27%) and only a few publications mentioned the exclusion of specific subgroups (e.g., Japanese cases [3/22] and co‐suspected pneumotoxic drugs [3/22]).

#### 3.2.2 Quality of discussion and bias considerations

Among the accessible articles (22/27), only four explicitly justified their query construction strategies, often referring to clinical judgement or consensus approaches aimed at balancing inclusiveness and specificity.^27^, ^30^, ^31^, ^33^ None of the accessible studies discussed their choice of the disproportionality analysis unit, nor did they address the temporal evolution of MedDRA coding and its impact on the reliability of analyses. Only one study justified restricting their dataset to reports from healthcare professionals to improve data validity.^23^ Country‐related biases were more frequently mentioned, with seven studies acknowledging the influence of regional differences in reporting behaviours and drug use, particularly highlighting Japan's disproportionate number of ILD reports and the implications for interpreting signal strength in East Asian populations.^21^, ^22^, ^25^, ^27^, ^30^, ^32^, ^33^ Three studies also noted limitations due to missing data and population heterogeneity across national databases.^22^, ^30^, ^32^

## 4 DISCUSSION

ADRs coding relies on the standardized MedDRA terminology. While this classification facilitates data harmonization, it also presents certain limitations—particularly the variability in its application over time, across countries, and among different types of reporters. Our study highlights this issue through an analysis of the PTs used within the SMQ for ILD. We observed a heterogeneous use of PTs, ranging from highly specific terms such as ‘pulmonary fibrosis’, ‘ARDS’, ‘organizing pneumonia’, and ‘immune‐mediated lung disease’, to more generic ones like ‘pulmonary toxicity’, ‘pneumonitis’, and ‘lung infiltration’. These broader terms may obscure the precise nature of the underlying pulmonary condition and emphasize the need for deeper analysis of pharmacovigilance narratives to improve case characterization. Furthermore, PTs explicitly related to ILD within the SMQ accounted for only a fraction of the total coded terms: 36.1% for amiodarone, 41.8% for everolimus, 40.1% for methotrexate, 36.5% for nivolumab, and 34.5% for pembrolizumab. The remaining PTs illustrate the diversity in coding practices and reflect the broad spectrum of pulmonary manifestations—an observation previously noted in analyses based on the French Pharmacovigilance Database.^34^ Several factors may contribute to this variability in PT coding within the ILD SMQ:

- Historical evolution of MedDRA coding practices

For older drugs such as amiodarone, methotrexate and everolimus, the distribution of coded PTs has remained relatively consistent over the past 20–24 years. Despite extensive literature linking these agents to ILD, this stability raises concerns about the effectiveness of awareness initiatives in pharmacovigilance. For more recent therapies such as nivolumab and pembrolizumab, we observed the emergence of the PT ‘immune‐mediated lung disease’ in the 2019–2020 period. Its use tripled for nivolumab and increased by 1.5‐fold for pembrolizumab between 2021 and 2022, aligning with growing recognition of immune‐related pulmonary events and the incorporation of this PT into MedDRA terminology. To our knowledge, only one prior study has examined the temporal evolution of ADR reporting in VigiBase, focusing on cutaneous reactions.^35^

- Country‐specific differences

Interestingly, USA and France showed the highest proportion of reports for amiodarone, likely reflecting its widespread use and known pulmonary toxicity. While Japan featured in the top five for all drugs, the proportion of Japanese reports did not dominate despite expectations based on known pharmacovigilance sensitivity. Although theoretically a given drug should exhibit a consistent AE profile regardless of the country, our findings reveal a clear divergence in coding practices. Coding patterns are similar between France and Japan but differ markedly from those observed in Germany, the UK, and the USA for all five studied drugs. Specifically, the term ‘interstitial lung disease’ predominates in reports from France and Japan, whereas more generic terms such as ‘pulmonary toxicity’ are more frequently used in Germany, the UK, and the USA. These observations are consistent with earlier reports from two decades ago,^4^ which suggested that national preferences in MedDRA term selection might stem from differences in clinical interpretation, documentation habits, or training in MedDRA use. A subsequent study in 2016^33^ examined geographical disparities in the coding of ILD‐related PTs within EudraVigilance, prompted by the disproportionately high number of cases reported in Japan. This study highlighted significant geographical variability and a distinct medical culture in Japan favouring the use of specific PT ‘ILD’. The authors hypothesized a possible detection bias related to heightened media attention following the gefitinib case, rather than a confirmed genetic predisposition. In fact, retrospective analyses of the gefitinib case in Japan have revealed significant flaws in regulatory and pharmacovigilance processes contributed to increase vigilance and reporting of ILD cases.^36^ The study underscored the need for coding harmonization to minimize geographical bias in disproportionality signal detection. More recently, a 2019 VigiBase study reaffirmed this Japanese coding preference.^37^ Thus, despite MedDRA's intended harmonization, these persistent coding differences reflect underlying cultural, clinical, and regulatory variations in the diagnosis and reporting of ILD.

- Reporter‐related differences

Across all studied drugs, healthcare professionals account for the majority of reports (average 75%). This disparity is likely due to regulatory obligations mandating reporting by certain professionals, established reporting habits within hospital departments, pharmacovigilance training. Notably, coding practices differ between patient and healthcare professionals. Patients and lay reporters tend to use broader, less specific terms such as ‘pneumonitis’, whereas healthcare professionals employ standardized and more precise medical terminology. These coding variations may reflect differences in medical knowledge and clinical experience. The PT ‘pulmonary fibrosis’ is more frequently used by nonhealthcare professionals for amiodarone, possibly because this term appears in patient information leaflets. The recently introduced PT ‘immune‐mediated lung disease’ was reported almost exclusively by healthcare professionals, reflecting its use in oncology and immunology contexts where diagnosis is more nuanced. This finding underscores that coding quality is closely linked to reporter expertise and highlights the critical need for rigorous oversight of coding derived from heterogeneous sources—particularly as pharmacovigilance databases and spontaneous reporting systems become increasingly accessible to patients. Pharmacovigilance databases analyses are increasingly utilized due to their apparent methodological accessibility. However, accurate interpretation requires a nuanced understanding of their inherent limitations.

Our systematic literature review reveals that biases related to coding—such as temporal changes, geographical differences, and reporter‐related variability—are rarely, if ever, acknowledged in publications employing these analyses. Moreover, sensitivity analyses, which are crucial to test the robustness of detected signals, especially for rare and diagnostically challenging AEs like interstitial lung disease, are largely absent. This lack of methodological reflexivity threatens the validity of conclusions drawn from such studies.

Although ILD was used here as a case example, the methodological issues identified—query construction strategies, choice of the disproportionality analysis unit, temporal evolution of MedDRA coding, country‐specific differences and reporter‐related differences— are likely to affect other ADRs as well. This includes, for instance, the SMQ ‘DRESS syndrome’ (which includes PTs reflecting symptoms such as angioedema, abnormal renal or hepatic laboratory findings, or skin manifestations) or the SMQ ‘hepatic disorders’ (which includes other SMQs, including ‘drug related hepatic disorders – comprehensive search’, containing PTs such as ‘hepatocellular injury’, ‘cholestatic liver injury’, ‘hepatitis toxic’, or ‘immune‐mediated hepatitis’,). Nonetheless, dedicated studies are needed to develop case‐by‐case guidance.

## 5 CONCLUSIONS

Pharmacovigilance is essential to detect rare, serious and delayed‐onset ADR, particularly when clinical diagnosis is challenging and specific biomarkers are lacking such as drug‐induced interstitial lung disease. Our analysis highlights the substantial variability in MedDRA coding practices across time, countries, and reporter types—an issue that critically affects the reliability of drug safety analyses, as inaccurate or inconsistent coding can distort signal detection. Although disproportionality analysis is a key component of statistical signal detection, not all signals of disproportionate reporting require further investigation, and conversely, some drug‐event combinations that do not appear as signals of disproportionate reporting may still warrant further review, as highlighted in the European Good Pharmacovigilance Practices (GVP), particularly Module IX on signal management.^38^ Additionally, it is important to recall that disproportionality analysis alone is insufficient to infer causality or support regulatory action. It should be complemented by established causality frameworks, such as the Bradford Hill criteria.

The potential of artificial intelligence (AI) to assist in ADR coding is promising and warrants consideration, particularly through automating the selection of the most appropriate MedDRA terms. However, AI systems could be trained on historically biased pharmacovigilance databases. Consequently, initiatives such as the VigiPoint natural language processing algorithm, if inadequately trained, risk perpetuating or even amplifying existing coding biases rather than mitigating them.^39^ Therefore, the deployment of AI in this context requires rigorously validated training datasets and ongoing expert pharmacovigilance supervision.

Careful selection of initial MedDRA terms for ILD coding is essential to avoid false‐positive or false‐negative disproportionality signals during analysis. Accordingly, we recommend several actions to enhance the robustness and interpretability of future pharmacovigilance studies involving ILD:

- Systematically and critically assess MedDRA query construction prior to conducting disproportionality analyses:
  ○ Provide a clear rationale for the choice of terms and the selected hierarchy level (e.g., Preferred Term, SMQ, or HLT).
- Systematically perform sensitivity analyses using alternative coding strategies:
  ○ These may include restricting analyses by:
    - excluding reports from countries with known divergent coding practices (e.g., Japan)
    - excluding reports submitted by nonhealthcare professionals
    - removing cases involving co‐suspected pneumotoxic drugs
    - removing reports listing an excessive number of AEs.
- Improve training in pharmacovigilance methodology, particularly for those performing disproportionality analyses:
  ○ Promote critical awareness of inherent coding biases and their potential influence on signal interpretation.

## AUTHOR CONTRIBUTIONS

**Romane Freppel, Jean‐Luc Faillie** and **Aurélie Grandvuillemin** contributed to the conception and design of the study, data analysis and interpretation, and manuscript writing. **Adeline Benis** and **Philippe Bonniaud** contributed to data interpretation.

## CONFLICT OF INTEREST STATEMENT

The authors declare no conflict of interest.

## Supporting information


**Table S1** List of Preferred Terms (PTs) included in the broad SMQ for Interstitial Lung Disease
**Table S3‐1** Main characteristics of patients with ILD associated with 5 drugs: comparison between cosuspect and sole suspect
**Table S3‐2** Co‐suspected drugs
**Table S3‐3** Reporter type distribution by drug in Preferred Terms of the SMQ Broad ‘ILD’ reports
**Figure S4** Type of MedDRA queries used in studies included in the systematic literature review

## Data Availability

The data used in this study were obtained from the global pharmacovigilance database VigiBase, which is accessible to all French Regional Pharmacovigilance Centers. The datasets generated and/or analysed during the current study are available from the corresponding author on request.

## References

[1] Skeoch S , Weatherley N , Swift AJ , et al. Drug‐induced interstitial lung disease: a systematic review. JCM. 2018;7(10):356. 30326612 10.3390/jcm7100356 PMC6209877

[2] Spagnolo P , Bonniaud P , Rossi G , Sverzellati N , Cottin V . Drug‐induced interstitial lung disease. Eur Respir J. 2022;60(4):2102776. doi:10.1183/13993003.02776-2021 35332071

[3] Matsuno O . Drug‐induced interstitial lung disease: mechanisms and best diagnostic approaches. Respir Res. 2012;13(1):39. doi:10.1186/1465-9921-13-39 22651223 PMC3426467

[4] Koo LC , Clark JA , Quesenberry CP , et al. National differences in reporting ‘pneumonia’ and ‘pneumonia interstitial’: an analysis of the WHO international drug monitoring database on 15 drugs in nine countries for seven pulmonary conditions. Pharmacoepidemiol Drug Saf. 2005;14(11):775‐787. doi:10.1002/pds.1071 15654720

[5] Tregunno PM , Fink DB , Fernandez‐Fernandez C , Lázaro‐Bengoa E , Norén GN . Performance of probabilistic method to detect duplicate individual case safety reports. Drug Saf. 2014;37(4):249‐258. 24627310 10.1007/s40264-014-0146-y

[6] Page MJ , McKenzie JE , Bossuyt PM , et al. The PRISMA 2020 statement: an updated guideline for reporting systematic reviews. BMJ. 2021;372:n71. 33782057 10.1136/bmj.n71 PMC8005924

[7] Matsumoto K , Nakao S , Hasegawa S , et al. Analysis of drug‐induced interstitial lung disease using the Japanese adverse drug event report database. SAGE Open Med. 2020;8:2050312120918264. doi:10.1177/2050312120918264 32528682 PMC7262990

[8] Sato J , Uchida M , Wakabayashi H , Shimizu T . Evaluation of lung toxicity related to the treatment with alectinib using a pharmacovigilance database. Anticancer Res. 2022;42(6):3109‐3116. 35641286 10.21873/anticanres.15799

[9] Kanbayashi Y , Kaneko Y , Kobayashi M , Wakabayashi H , Shimizu T , Uchida M . Evaluation of lung adverse events associated with lenvatinib: a post‐marketing surveillance study. In Vivo. 2025;39(1):346‐352. doi:10.21873/invivo.13834 39740919 PMC11705116

[10] Kozaru M , Kambara H , Higuchi A , Kagatsume T , Hosohata K . Association of clopidogrel with interstitial lung disease: gaining insight through the Japanese pharmacovigilance database. Vasc Health Risk Manag. 2024;20:415‐420. doi:10.2147/VHRM.S482190 39247557 PMC11378778

[11] Oura K , Tanaka M , Matsumoto K , et al. Analysis of drug‐induced interstitial lung disease caused by herbal medicine using the Japanese adverse drug event report database. BMC Complement Med Ther. 2024;24(1):121. doi:10.1186/s12906-024-04428-y 38486172 PMC10938654

[12] Oshima Y , Tanimoto T , Yuji K , Tojo A . EGFR–TKI‐associated interstitial pneumonitis in nivolumab‐treated patients with non–small cell lung cancer. JAMA Oncol. 2018;4(8):1112. 29327061 10.1001/jamaoncol.2017.4526 PMC5885195

[13] Raschi E , Fusaroli M , Diemberger I , Poluzzi E . Direct oral anticoagulants and interstitial lung disease: emerging clues from pharmacovigilance. Drug Saf. 2020;43(11):1191‐1194. doi:10.1007/s40264-020-00990-9 32845443 PMC7447592

[14] Raschi E , Fusaroli M , Ardizzoni A , Poluzzi E , De Ponti F . Cyclin‐dependent kinase 4/6 inhibitors and interstitial lung disease in the FDA adverse event reporting system: a pharmacovigilance assessment. Breast Cancer Res Treat. 2021;186(1):219‐227. doi:10.1007/s10549-020-06001-w 33150548 PMC7641870

[15] Goldman, SA . Adverse event reporting and standardized medical terminologies: strengths and limitations. Drug Inf J avr 2002;36(2):439–444.

[16] Wu B , Shen P , Yin X , et al. Analysis of adverse event of interstitial lung disease in men with prostate cancer receiving hormone therapy using the Food and Drug Administration adverse event reporting system. Br J Clin Pharmacol. 2023;89(2):440‐448. 35349180 10.1111/bcp.15336

[17] Zhao M , Liu S , Xie R , Zhang J , Li J . Interstitial lung disease risk of anaplastic lymphoma kinase tyrosine kinase inhibitor treatment of non‐small cell lung cancer: a real‐world pharmacovigilance study. Expert Opin Drug Saf. 2023;22(12):1309‐1316. 37551674 10.1080/14740338.2023.2245324

[18] Zhang J , Qiu T , Zhou Y , Wu S , Chen E . Tyrosine kinase inhibitors‐associated interstitial lung disease used in non‐small cell lung cancer: a pharmacovigilance analysis based on the FDA adverse event reporting system database. Expert Opin Drug Saf. 2023;22(9):849‐856. 37026465 10.1080/14740338.2023.2193392

[19] Shi J , Liu X , Wu L , Jiang Y , Zhang Y , Wang Y . Interstitial lung disease with antibody–drug conjugates: a real‐world pharmacovigilance study based on the FAERS database during the period 2014–2023. Ther Adv Respir Dis. 2024;18:17534666241299935. doi:10.1177/17534666241299935 39660786 PMC11635890

[20] Lerman TT , Gadot C , Greenberg N , et al. The safety profile of amiodarone among older adults (age ≥75 years): a pharmacovigilance study from the FDA data. Am J Med. 2025;138(5):819‐826. 39842538 10.1016/j.amjmed.2025.01.011

[21] Lin W , Xu J , Liao Y , Lin X , Yang J , Zhuang W . Assessing safety concerns of interstitial lung disease associated with antibody‐drug conjugates: a real‐world pharmacovigilance evaluation of the FDA adverse event reporting system. Int J Clin Pharmacol. 2024;46(3):614‐622. 10.1007/s11096-023-01673-y 38100054

[22] Jiang T , Su H , Xu J , et al. Drug‐induced interstitial lung disease: a real‐world pharmacovigilance study of the FDA adverse event reporting system from 2004 to 2021. Therapeutic Advances in Drug Safety. 2024;15:20420986231224227. doi:10.1177/20420986231224227 38293566 PMC10823853

[23] Shi J , Liu X , Gao M , et al. Adverse event profiles of EGFR‐TKI: network meta‐analysis and disproportionality analysis of the FAERS database. Front Pharmacol. 2025;16:1519849. doi:10.3389/fphar.2025.1519849/full 40135231 PMC11933087

[24] Yuan J , Li Z , Ye M , Fu Z . A disproportionality analysis of interstitial lung disease associated with drug therapy in spontaneous adverse event reports. Expert Opin Drug Saf. 2025;17:1‐9. 10.1080/14740338.2025.2494689 40232264

[25] Wang H , Chen K , Ma S , et al. PPIs effect in EGFR‐TKI‐associated interstitial lung diseases in patients with non‐small cell lung cancer. BMC Cancer. 2025;25(1):263. doi:10.1186/s12885-025-13673-4 39953451 PMC11827195

[26] Mo Y , Qin Y , Li P , Wu M , Yu J , Chen D . Thyroxine alleviates interstitial lung disease induced by combined radiotherapy and immunotherapy. Cancer Lett. 2025;615:217504. 39880326 10.1016/j.canlet.2025.217504

[27] Reese SW , Cone E , Marchese M , et al. Lessons from pharmacovigilance: pulmonary immune‐related adverse events after immune checkpoint inhibitor therapy. Lung. 2021;199(2):199‐211. 33616727 10.1007/s00408-021-00425-x

[28] Ma Z , Zhang Y , Zhu M , Feng L , Zhang Y , An Z . Interstitial lung disease associated with anti‐HER2 anti‐body drug conjugates: results from clinical trials and the WHO'S pharmacovigilance database. Expert Rev Clin Pharmacol. 2022;15(11):1351‐1361. 36111954 10.1080/17512433.2022.2121705

[29] Ma Z , Pei J , Zhang Y , et al. Interstitial pneumonitis associated with EGFR/ALK tyrosine kinase inhibitors used in non–small cell lung cancer: an observational, retrospective, pharmacovigilance study. Expert Opin Drug Saf. 2023;22(3):237‐242. 35924402 10.1080/14740338.2022.2110235

[30] Lee MT , Lee JW , Lee HJ , et al. Interstitial lung disease following COVID‐19 vaccination: a disproportionality analysis using the global scale Pharmacovigilance database (VigiBase). BMJ Open Respir Res. 2023;10(1):e001992. 10.1136/bmjresp-2023-001992 PMC10729117 38081769

[31] Givry F , Lebargy F , Lebrun D , et al. Lung toxicity related to trimethoprim/sulfamethoxazole: pharmacovigilance data review. J Antimicrob Chemother. 2025;80(4):1148‐1152. 39989446 10.1093/jac/dkaf050

[32] Minagi A , Nawa H , Goda M , et al. Evaluation of interstitial lung disease complications caused by biologic agents using a spontaneous adverse drug reaction reporting database. Pharmacology Res & Perspec. 2025;13(2):e70063. doi:10.1002/prp2.70063 PMC11845275 39984304

[33] Pinheiro L , Blake K , Januskiene J , Yue Q , Arlett P . Geographical variation in reporting interstitial lung disease as an adverse drug reaction: findings from an European medicines agency analysis of reports in EudraVigilance. Pharmacoepidemiology and Drug. 2016;25(6):705‐712. 10.1002/pds.3998 27004571

[34] Yavordios S , Beltramo G , Freppel R , et al. Diffuse lung diseases ascribed to drugs: a nationwide observational study over 37 years using the French Pharmacovigilance database. Eur Respir J. 2025;65(2):2400756. 39510554 10.1183/13993003.00756-2024

[35] Strumia M , Perrin ML , Patras De Campaigno E , et al. Dermatological adverse drug reactions of anticancer drugs: international data of pharmacovigilance: VigiBase®. Therapies. 2022;77(2):219‐227. 10.1016/j.therap.2021.12.006 34973824

[36] Nishimura T , Tada H , Nakagawa M , Teramukai S , Matsui S , Fukusshima M . Lessons from gefitinib‐induced interstitial lung disease in Japan: problems in approval, pharmacovigilance, and regulatory decision‐making procedures. Pharmacy Pract (Granada Ed Impr). 2006;4(4):168‐178. doi:10.4321/s1885-642x2006000400004 PMC4155619 25214906

[37] Wakao R , Taavola H , Sandberg L , et al. Data‐driven identification of adverse event reporting patterns for Japan in VigiBase, the WHO global database of individual case safety reports. Drug Saf. 2019;42(12):1487‐1498. 31559542 10.1007/s40264-019-00861-y PMC6858382

[38] European Medicines Agency . Guideline on good pharmacovigilance practices (GVP)—Module IX: Signal management (Rev. 1) (Ref. No. EMA/827661/2011 Rev 1) 2017.

[39] Erlanson N , China JF , Taavola H , Norén GN . Clinical relatedness and stability of vigiVec semantic vector representations of adverse events and drugs in pharmacovigilance. Drug Saf. 2025;48(4):401‐413. 39833656 10.1007/s40264-024-01509-2 PMC11903574

