# Supplementary material for: Analysis of interstitial lung disease in pharmacovigilance databases: Coding challenges and interpretation biases—An update
Source: Br J Clin Pharmacol. 2026 Feb 15;92(7):2147–61. doi: 10.1002/bcp.70483 (PMC13304250; doi:10.1002/bcp.70483)
Supplement: Supplementary file 1 — Table S1 List of Preferred Terms (PTs) included in the broad SMQ for Interstitial Lung Disease Table S3‐1 Main characteristics of patients with ILD associated with 5 drugs: comparison between cosuspect and sole suspect Table S3‐2 Co‐suspected drugs Table S3‐3 Reporter type distribution by drug in Preferred Terms of the SMQ Broad ‘ILD’ reports Figure S4 Type of MedDRA queries used in studies included in the systematic literature review [file BCP-92-2147-s001.docx]

**Analysis of interstitial lung disease in pharmacovigilance databases : coding challenges and interpretation biases – an update**

Supplementary Material

S1: Broad SMQ

**Table S1** List of Preferred Terms (PTs) included in the broad SMQ for Interstitial Lung Disease

| Small airways disease | Eosinophilia myalgia syndrome |
| --- | --- |
| Alveolitis necrotising | Pulmonary toxicity |
| Radiation alveolitis | Pulmonary vasculitis |
| Alveolitis | Restrictive pulmonary disease |
| Interstitial lung abnormality | Biopsy lung abnormal |
| Respiratory syncytial virus bronchiolitis | Pulmonary bulla |
| Necrotising bronchiolitis | Airway remodelling |
| Obliterative bronchiolitis | Complications of transplanted lung |
| Bronchiolitis | Pulmonary contusion |
| Radiation bronchitis | Allergic eosinophilia |
| Low lung compliance | Pulmonary eosinophilia |
| Diffuse alveolar damage | Pulmonary septal thickening |
| Pleuroparenchymal fibroelastosis | Granulomatosis with polyangiitis |
| Idiopathic pulmonary fibrosis | Pulmonary granuloma |
| Progressive massive fibrosis | Pulmonary alveolar haemorrhage |
| Radiation fibrosis - lung | Pulmonary haemosiderosis |
| Pulmonary fibrosis | Langerhans' cell histiocytosis |
| Eosinophilic granulomatosis with polyangiitis | Lung induration |
| Lung infiltration | Acute lung injury |
| Transfusion-related acute lung injury | Lymphangioleiomyomatosis |
| Confirmed e-cigarette or vaping product use associated lung injury | Cystic lung disease |
| Probable e-cigarette or vaping product use associated lung injury | Polyarteritis nodosa |
| Chronic graft versus host disease in lung | Organising pneumonia |
| Immune-mediated lung disease | Pneumonitis chemical |
| Alveolar lung disease | Granulomatous pneumonitis |
| Autoimmune lung disease | Lupus pneumonitis |
| Pulmonary necrosis | Mixed obstructive and restrictive lung disease |
| Lung opacity | Rheumatoid lung |
| Acute interstitial pneumonitis | Systemic sclerosis pulmonary |
| Idiopathic interstitial pneumonia | Lung transplant rejection |
| Eosinophilic pneumonia acute | Pulmonary bullae rupture |
| Eosinophilic pneumonia chronic | Pulmonary sarcoidosis |
| Eosinophilic pneumonia | Sarcoidosis |
| Hypersensitivity pneumonitis | Acute respiratory distress syndrome |
| Pneumonitis | Goodpasture's syndrome |
| Connective tissue disease-associated interstitial lung disease | Toxic oil syndrome |
| Childhood interstitial lung disease | Loefgren syndrome |
| Interstitial lung disease | Antisynthetase syndrome |
| Radiation pneumonitis | Pulmonary renal syndrome |
| Pulmonary radiation injury |  |
| Alveolar proteinosis |  |
| Bronchiolitis obliterans syndrome |  |
| Idiopathic pneumonia syndrome |  |
| Combined pulmonary fibrosis and emphysema |  |

S2: Duplicate detection method

Exclusion criteria included:

- PTs unrelated to ADR
- Duplicate reports, identified throughe a two-step process :
  - First potentiel duplicates cases were flagged using the VigiMatch algorithm implemented in VigiBase. Then, a manuel review was performed to validate or discard suspected duplicates based on the following matching criteria:
    - Age (±1 year)
    - Sex
    - Reporter identity
    - Onset date of adverse event
    - Identical MedDRA term (listed twice for emphasis)
    - Reporting country

If discrepancies were present in any of these six variables, cases were not considered duplicates—unless:

- Different MedDRA terms or onset dates were reported by different reporter types (non-healthcare professional reports were excluded)
- Reports came from different countries; in such cases, reports from national pharmacovigilance centers were prioritized over those submitted by pharmaceutical companies from abroad

When duplication was still suspected, preference was given to the report with the higher completeness score and/or more recent submission date, provided all other factors were equal.

S3: Descriptive analysis of included ILD ICSRs

**Table S3-1** Main characteristics of patients with ILD associated with 5 drugs: comparison between cosuspect and sole suspect

| Characteristics  (n,%) | Amiodarone | | | Everolimus | | | | | Méthotrexate | | | | | Nivolumab | | | Pembrolizumab | | | | | |  |  |  |  |  |  |  |  |  |  |  |  |  |
| --- | --- | --- | --- | --- | --- | --- | --- | --- | --- | --- | --- | --- | --- | --- | --- | --- | --- | --- | --- | --- | --- | --- | --- | --- | --- | --- | --- | --- | --- | --- | --- | --- | --- | --- | --- |
|  | **Sole** **suspect** | **Cosuspect** | | **Sole** **suspect** | | | **Cosuspect** | | **Sole** **suspect** | | | **Cosuspect** | | **Sole** **suspect** | | **Cosuspect** | **Sole** **suspect** | | | **Cosuspect** | | |  |  |  |  |  |  |  |  |  |  |  |  |  |
|  | **N=6,268** | | | **N= 3,688** | | | | | **N=6,183** | | | | | **N=4,831** | | | **N=3,833** | | | | | |  |  |  |  |  |  |  |  |  |  |  |  |  |
|  | **n = 4491 (71.6)** | **n = 1777 (28.4)** | | **n = 2339 (63.4)** | | | **n = 1349 (36.6)** | | **n = 2652 (43,0)** | | | **n = 3531 (57,1%)** | | **n = 2940 (60.9%)** | | **n = 1891 (39.1%)** | **n = 2607 (68,0%)** | | | **n = 1226 (32,0%)** | | |  |  |  |  |  |  |  |  |  |  |  |  |  |
| Male  Female  Not specified | 2,947 (65.6)  1,385 (30.8)  159 (3.5) | 1,180 (66.4)  555 (31.2)  42 (2.4) | | 693 (29.6)  1,449 (61.9)  197 (8.4) | | | 415 (30.8)  864 (640)  70 (0.1) | | 905 (34.1)  1,603 (60.4)  144 (5.4) | | | 1,118 (31.7)  2,167 (61.4)  246 (7.0) | | 2,003 (68.1)  699 (23.8)  238 (8.1) | | 1,156 (61.1)  570 (30.1)  165 (8.7) | | | 1,772 (68.0)  718 (27.5)  116 (4.4) | | 708 (57.7)  425 (34.6)  93 (7.6) | | | |  | |  |  |  |  |  |  |  |  |  |
|  |  |  | | |  | | |  | |  | | |  | |  | | |  | | |  | | | | |  | | | |  | |  |  |  |  |
| Age (n,%)  Mean  Median (Q1 ; Q3)  Min ; Max | 73.5  75 (68 ; 80)  1 ; 99 | 71.9  74 (67 ; 80)  3 ; 94 | | 62.7  64 (56 ; 71)  0 ; 94 | | | 62.4  64 (56 ; 71)  0 ; 100 | | 65.2  67 (59 ; 75)  3 ; 95 | | | 58.4  63 (52 ; 71)  0 ; 94 | | 65.5  68 (61 ; 74)  0 ; 94 | | 63.2  65 (57 ; 72)  6 ; 89 | 67.0  69 (61 ; 75)  5 ; 103 | | | 65.4  67 (59 ; 73)  4 ; 100 | | |  | | |  |  |  |  |  |  |  |  |  |  |
|  | n= 3,809  (60.8%) | n=1,153  (18.4) | | n= 1,444  (39.2) | | | n= 922  (25) | | n= 2,276  (36,8) | | | n= 2,274  (36,8) | | n= 2,297 (78.1) | | n= 1,502 (79.4) | | n= 2139  (82.0) | | | n= 932  (76.0) |  | | | | | |  | | |  | |  |  |  |
|  |  | |  | | |  | | | | |  | | | | |  | | | | |  | | |  | | | | |  | | | |  |  |  |

**Table S3-2** Co-suspected drugs

|  | Amiodarone | | Everolimus | | Methotrexate | | Nivolumab | | Pembrolizumab | |
| --- | --- | --- | --- | --- | --- | --- | --- | --- | --- | --- |
| Cosuspect drugs | Apixaban  Bisoprolol  Rivaroxaban  Fluindione  Furosémide  Nivolumab  Digoxine  Simvastatine | 20 (1,0%)  18 (0,9%)  17 (0,8%)  14 (0,7%)  14 (0,7%)  11 (0,5%)  10 (0,5%)  10 (0,5%) | Ex**e**mestan  Letrozole  Octréotide  Ciclosporin  Prednisone  Clinical trials  Mycophénolate  Lenvatinib | 139 (9.4%)  22 (1.5%)  19 (1.3%)  14 (1.0%)  13 (0.9%)  11 (0.7%)  10 (0.7%)  9 (0.6%) | Etanercept  Infliximab  Adalimumab  Luflunomide  Prednisone  Rituximab  Tocilizumab  Abatacept | 251 (6.5%)  212 (5.5%)  202 (5.2%)  125 (3.2%)  107 (2.8%)  72 (1.9%)  59 (1.5%)  54 (1.4%) | Ipilimumab  Osimertinib  Azacitidine  Docetaxel  Prednisolone  Paclitaxel  Pembrolizumab  Carboplatine | 503 (26.0%)  37 (1.9%)  28 (1.5%)  26 (1.3%)  23 (1.2%)  17 (0.9%)  16 (0.8%)  10 (0.5%) | Pemetrexed  Carboplatine  Lenvatinib  Ipilimumab  Paclitaxel  Pemetrexed  Cisplatine  Nivolumab | 76 (5,8%)  71 (5.4%)  40 (3.1%)  24 (1.8%)  24 (1.8%)  16 (1.2%)  16 (1.2%)  16 (1,2%) |

OMIC, Other medically important condition; ARDS, Acute respiratory distress syndrome IPF, Idiopathic pulmonary fibrosis; HAP, Pulmonary alveolar haemorrhage

**Table S3-3** Reporter type distribution by drug in Preferred Terms of the SMQ Broad « ILD » reports

| Characteristics | Amiodarone | | Everolimus | | Methotrexate | | Nivolumab | | Pembrolizumab | |
| --- | --- | --- | --- | --- | --- | --- | --- | --- | --- | --- |
|  | Non-healthcare professionals | Healthcare professionals | Non-healthcare professionals | Healthcare professionals | Non-healthcare professionals | Healthcare professionals | Non-healthcare professionals | Healthcare professionals | Non-healthcare professionals | Healthcare professionals |
| SMQ ILD PT | **n = 1706 (27.3%)** | **n = 4541 (72,7%)** | **n = 791 (20,8%)** | **n = 3013 (79,2%)** | **n = 1462 (24,1%)** | **n = 4600 (75,9%)** | **n =1424 (29,1%)** | **n = 3471 (70,9%)** | **n = 822 (21,0%)** | **n = 3099 (79,0%)** |
| MedDRA Terms  Interstitial lung disease  Pulmonary fibrosis  Pulmonary toxicity  Pneumonitis  Lung infiltration  ARDS  Organising pneumonia  Sarcoidosis  Hypersensitivity pneumonitis  Rheumatoid lung  Obliterative bronchiolitis  Lung opacity  Immune-mediated lung disease  Other | 298 (17,5%)  545 (32,0%)  471 (27,6%)  88 (5,2%)  43 (2,5%)  74 (4,3%)  58 (3,4%)  3 (0,2%)  3 (0,2%)    0  4 (0,2%)  7 (0,4%)  0  112 (6,6%) | 1,800 (39,6%)  1,003 (22,1%)  324 (71%)  378 (8,3%)  245 (5,4%)  197 (4,3%)  105 (2,3%)  3 (0,1%)  61 (1,3%)  0  8 (0,2%)  27 (0,6%)  0  390 (8,6%) | 175 (22,1%)  16 (2,0%)  30 (3,8%)  419 (52,8%)  36 (4,6%)  19 (2,4%)  29 (3,7%)  2 (0,3%)  4 (0,5%)  0  3 (0,4%)  0  0  58 (7,3%) | 1,392 (46,2%)  48 (1,6%)  39 (1,3%)  1,156 (38,4%)  130 (4,3%)  34 (1,1%)  36 (1,2%)  1 (<0.1%)  25 (0,8%)  0  5 (0,2%)  0  0  183 (6,1%) | 376 (25,7%)  297 (20,3%)  77 (5,3%)  244 (16,7%)  47 (3,2%)  105 (7,2%)  26 (1,8%)  39 (2,7%)  33 (2,3%)  29 (2,0%)  26 (1,8%)  11 (0,8%)  0  152 (10,4%) | 1,948 (42,4%)  620 (13,5%)  123 (2,7%)  736 (16,0%)  207 (4,5%)  230 (5,0%)  101 (2,2%)  55 (1,2%)  90 (2,0%)  34 (0,7%)  13 (0,3%)  43 (0,9%)  0  400 (8,7%) | 107 (7,5%)  17 (1,2%)  19 (1,3%)  898 (63,1%)  12 (0,8%)  23 (1,6%)  17 (1,2%)  27 (1,9%)  4 (0,3%)  0  0  7 (0,5%)  238 (16,7%)  55 (3,9%) | 1,685 (49,0%)  21 (0,6%)  24 (0,7%)  1,150 (33,1%)  26 (0,8%)  30 (0,9%)  148 (4,3%)  61 (1,8%)  23 (0,7%)  0  6 (0,2%)  6 (0,2%)  125 (3,6%)  166 (4,8%) | 36 (4,4%)  11 (1,3%)  22 (2,7%)  494 (60,1%)  7 (0,9%)  9 (1,1%)  23 (2,8%)  32 (4,0%)  5 (0,6%)  0  0  13 (1,6%)  124 (15,1%)  59 (7,2%) | 1,330 (43,0%)  50 (1,6%)  23 (0,7%)  1,052 (34,0%)  29 (0,9%)  39 (1,3%)  93 (3,0%)  52 (1,7%)  21 (0,7%)  0  3 (0,1%)  32 (1,0%)  210 (6,8%)  165 (5,3%) |

ARDS, Acute respiratory distress syndrome

S4: Literature review


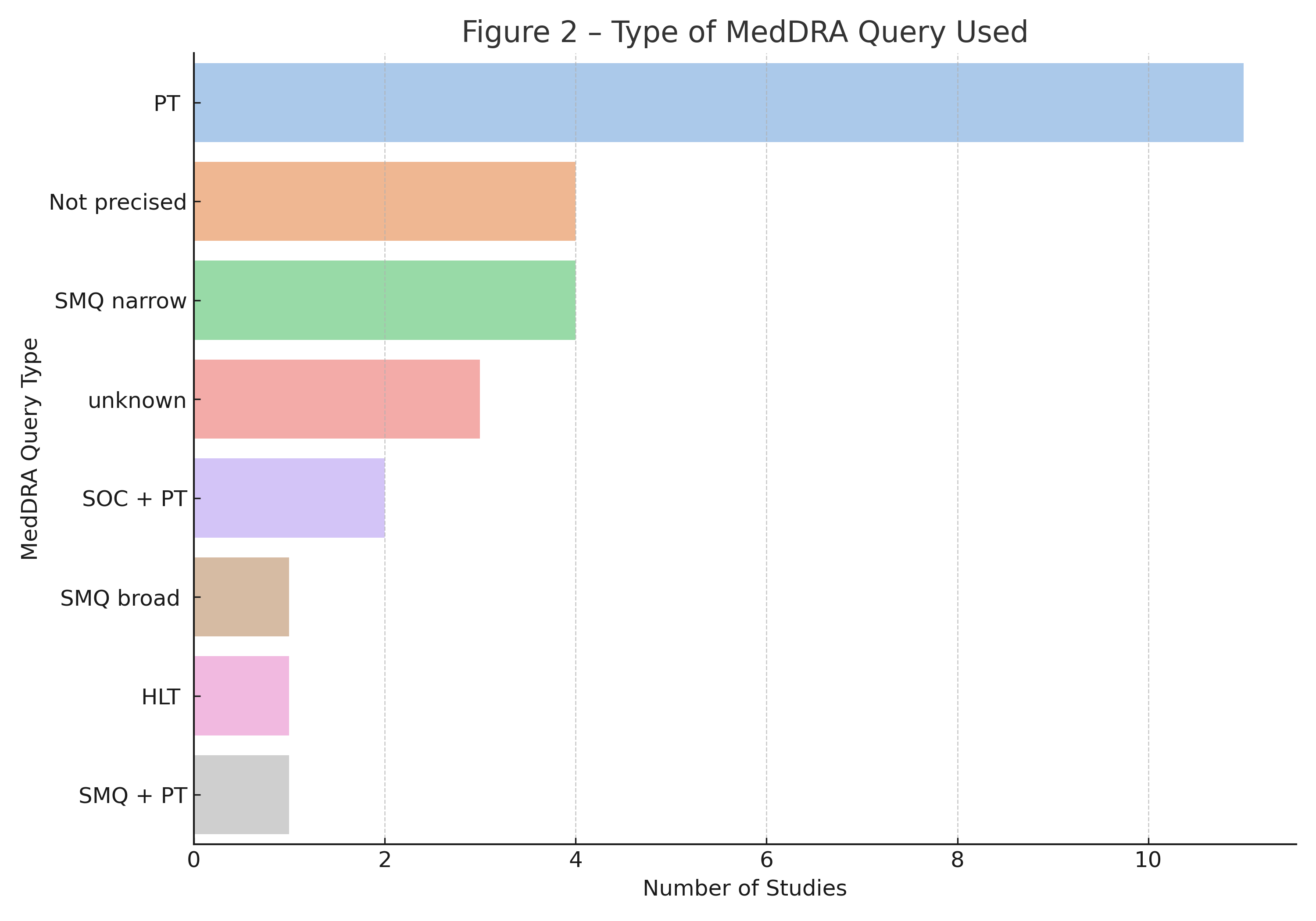


**Fig. S4** Type of MedDRA queries used in studies included in the systematic literature review
